# Modeling the transition from acute to chronic postsurgical pain in youth: A narrative review of epidemiologic, perioperative, and psychosocial factors

**DOI:** 10.1080/24740527.2022.2059754

**Published:** 2022-06-09

**Authors:** Brittany N. Rosenbloom, Joel Katz

**Affiliations:** a Child Health Evaluative Sciences, The Hospital for Sick Children, Toronto, Ontario, Canada; b Department of Psychology, York University, Toronto, Ontario, Canada

**Keywords:** pediatric, chronic postsurgical pain, anxiety, fear avoidance, parents, siblings, peers

## Abstract

A growing number of studies have identified high rates of pediatric chronic postsurgical pain (CPSP) after major surgery. Pediatric CPSP is associated with pain-related distress and comorbid mental health outcomes, such as anxiety and depression. From a biopsychosocial perspective, youth factors, such as genetics, epigenetics, sex, presurgical pain, sleep, anxiety, and pain catastrophizing, as well as parent factors, such as cognitive appraisals of their child’s pain expression and pain catastrophizing, converge and lead to chronic pain disability. A comprehensive and testable psychosocial model of the transition from acute to chronic pediatric postsurgical pain has not been developed. This narrative review begins by evaluating the epidemiology and trajectories of pediatric CPSP and moves on to examine the more influential psychosocial models that have been proposed to understand the development of pediatric CPSP. Much of the literature to date has been conducted on adolescents undergoing spinal fusion. To conceptualize the transition from acute to chronic pain in youth, a combined diathesis-stress and interpersonal fear avoidance model is presented. Novel areas of future research include the potential influence that siblings and peers have on a youth’s development of CPSP as well as the influence of gender.

## RÉSUMÉ

Un nombre croissant d'études ont recensé des taux élevés de douleur post-chirurgicale chronique pédiatrique après une intervention chirurgicale majeure. La douleur post-chirurgicale chronique pédiatrique est associée à de la détresse liée à la douleur et à des problèmes de santé mentale concomitants tels que l'anxiété et la dépression. D'un point de vue biopsychosocial, les facteurs liés aux jeunes tels que la génétique, l'épigénétique, le sexe, la douleur pré-chirurgicale, le sommeil, l'anxiété et la catastrophisation de la douleur, et les facteurs parentaux tels que l'évaluation cognitive de l'expression de la douleur et la catastrophisation de la douleur chez leur enfant, convergent et mènent à une invalidité attribuable à la douleur chronique. Aucun modèle psychosocial complet et vérifiable de la transition de la douleur post-chirurgicale pédiatrique aigue à la douleur post-chirurgicale chronique n'a encore été élaboré. Cette revue narrative commence par l'évaluation de l'épidémiologie et des trajectoires de douleur post-chirurgicale pédiatrique chronique et poursuit en examinant les modèles psychosociaux les plus influents qui ont tenté de comprendre le développement de la douleur post-chirurgicale chronique pédiatrique. Une grande partie de la littérature à ce jour a poré sur les adolescents subissant une fusion vertébrale. Afin de conceptualiser la transition de la douleur aigue à la douleur chronique chez les jeunes, un modèle combinant le diathèse-stress et l'évitement de la peur interpersonnelle est présenté. Parmi les domaines de recherche novateurs, on compte l'influence potentielle que les frères et sœurs et les pairs ont sur le développement de la douleur post-chirurgicale chronique chez les jeunes, ainsi que l'influence du genre.

The number of studies on the transition from acute to chronic postsurgical pain has grown over the past decade, making it apparent how many children and adolescents develop chronic postsurgical pain (CPSP). CPSP is defined as pain that develops, or increases in intensity, after a surgical procedure; is a continuation of acute postsurgical pain or develops after an asymptomatic period; is localized to the surgical site or projected to a referred area; persists for at least 3 months; affects quality of life; and is not caused by other factors.^1^ This narrative review evaluates the epidemiology and trajectories of pediatric CPSP as well as the more influential psychosocial models that have been proposed to understand the development of pediatric CPSP. Based on recent findings in the field, we present a combined diathesis-stress and interpersonal fear avoidance model to conceptualize the transition from acute to chronic pain.

## Search Strategy

A search of the literature was conducted through the PubMed database using the following words in English: “postoperative pain OR postsurgical pain” AND “children OR adolescents OR youth” AND “persistent OR chronic.” We included studies that (1) prospectively evaluated postsurgical outcomes following major surgery and (2) had a measurement of pain with a validated tool presurgically as well as at least 3 months postsurgically. Non-English-language studies were excluded. Studies that included youth with cancer or who had limb salvage surgeries were excluded as well.

## Epidemiology of Pediatric Chronic Postsurgical Pain

Table 1 shows the incidence of chronic postsurgical pain reported by prospective studies, highlighting the type of surgery, sample size, and definition of CPSP used by the authors. The earliest study evaluating the incidence of chronic postsurgical pain was conducted by Landman et al.^2^ on patients undergoing spinal fusion surgeries for idiopathic scoliosis, one of the more common surgeries among adolescents. Since 2011, eight other studies have reported on the incidence of chronic postsurgical pain in children, with half of them examining orthopedic and general surgeries combined and the other half focused on spinal fusion surgery. The 12-month incidence of chronic postsurgical pain ranges from 11% to 54%, which is similar to the adult literature.^3^ However, unlike the adult literature, there appears to be variability between scoliosis surgeries and other types of major surgeries in terms of pain outcomes. This difference may be an artifact of the variability in the criteria used to determine the presence of CPSP by pediatric studies (e.g., a score of moderate or severe on the Scoliosis Research Scale–30^4^ or a score of one or more on the visual analogue scale [VAS] or numeric rating scale [NRS] for pain). When comparing the most common criterion for chronic postsurgical pain (i.e., a score of four or more on a 0–10 NRS), the rates differ by approximately 20% between 19.1%^5^ and 418%^6^ 1 year after surgery. Nevertheless, even when one uses the most conservative criteria to determine CPSP, the 1-year incidence of pediatric chronic postsurgical pain is high and concerning. Similar to research conducted in adults, very few prospective studies follow children who have undergone major surgery for longer than 1 year. However, the two exceptions are Landman et al.,^2^ in which 52% of patients reported mild to severe pain 2 years after spinal fusion, and Sieberg et al.,^7^ who reported an incidence of ~35% for moderate or severe pain 5 years after spinal fusion. These longer-term statistics are alarming and highlight the need for improved understanding and management of pediatric CPSP.

**Table 1.** Incidence of CPSP in children and adolescents following various surgical procedures.

Study	Surgical procedure	No. of patients (age range of patients in years)	Follow-up time (months)	Incidence of CPSP (%)	Pain intensity criterion for presence of CPSP
Landman et al.^2^	Spinal fusion	295 (8–22)	12	53.6	Mild to severe range on the SRS-30
			24	53.2	
Pagé et al.^11^	Spinal fusion	83 (8–18)	6	23	Pain rated as ≥4 on a 0–10 NRS
	Osteotomy		12	22	
	Ravitch				
	Nuss				
	Laparotomy				
	Thoracotomy				
Sieberg et al.^7^	Spinal fusion	190 (8–21)	12	11	Moderate-to-severe range on the SRS-30 in the past month
Rabbitts et al.^12^	Major orthopedic surgery	60 (10–18)	4	18.3	Late pain recovery trajectory
Batoz et al.^8^	Orthopedics	291 (6–18)	3	10.9	Pain >2 on 100 mm VAS
	Thoracic				
	Laparotomy/laparoscopy				
	Uro/inguinal				
Chidambaran et al.^6^	Spinal fusion	144 (10–18)	2–3	37.8	Pain >3/10 on NRS
			12	41.8	
Julien-Marsollier et al.^9^	Posterior fixation spinal surgery	36 (mean 15, SD 2)	12	52.8	Pain >3/10 on NRS
Perello et al.^5^	Surgical posterior vertebral fusion	48 (10–18)	6	19.05	Pain >0/10 on NRS
Rosenbloom et al.^13^	Spinal fusion	264 (8–18)	6	35.5	Pain ≥4/10 on NRS
	Osteotomy		12	38.7	
	Ravitch				
	Nuss				
	Laparotomy				
	Thoracotomy				
Ocay et al.^15^	Spinal fusion	106 (mean 15, SD 2)	6	60.4	Moderate-to-severe range on the SRS-30 in the past month

In terms of the nature of pediatric CPSP, Batoz et al.^8^ reported that 11% of children had moderate-to-severe pain 3 months after surgery and a total of 7% had pain of neuropathic origin, assessed by the Douleur Neuropathique (DN4). Additionally, in a study of 36 children undergoing surgery for idiopathic scoliosis, 47% had pain of neuropathic origin, assessed through the DN4, 1 year after surgery.^9^ To our knowledge, no other studies have reported on the incidence of postsurgical neuropathic pain. Importantly, however, the two studies did not physically examine the participants and instead relied on a questionnaire to determine the presence of neuropathic pain. Given the apparently high incidence of neuropathic pain following surgery for scoliosis, more accurate measurement of neuropathic pediatric postsurgical pain is required, most notably by physical examination and using a validated scale such as the Pediatric PainSCAN.^10^

A recent modification to the definition of SPSP requires that the pain negatively affect quality of life.^1^ Pagé et al.,^11^ Rabbitts et al.,^12^ Batoz et al.,^8^ Chidambaran et al.,^6^ and Rosenbloom et al.^13^ used measures of disability, functioning, or activity, all finding a significant positive association between pain intensity and functional disability. For example, Rabbitts et al.^12^ showed that children with a late pain recovery trajectory had lower quality of life and greater activity limitations than those with an early recovery. Batoz et al.^8^ found that of those children with moderate-to-severe postsurgical pain, 78% reported moderate interference with activities and 22% reported severe interference with activities. Using the Functional Disability Index (FDI), Chidambaran et al.^6^ reported significantly greater disability in children classified as having chronic pain up to 12 months after surgery than those children classified as not having chronic pain. Using the FDI, Rosenbloom et al.^13^ found that 18% and 14% had both moderate-to-severe pain and moderate-to-severe disability at 6 and 12 months after surgery, respectively. In contrast, Pagé et al.^11^ found low levels of disability in the entire sample 12 months after surgery using the FDI (i.e., even among participants who reported moderate-to-severe pain intensity).

Though functional disability must be included in studies of chronic postsurgical pain, it should be evaluated within the context of the type of surgery and the clinical recommendations for return to regular activity. For instance, with more invasive surgeries, such as spinal fusion for scoliosis, it is recommended that children return to their typical level of activity 6 months after surgery. In contrast, children who undergo hernia repair typically return to normal activity within weeks of their surgery.

## Postsurgical Pain Trajectories

Evaluating the incidence of pain and disability is one metric, however, and, similar to the adult literature,^14^ pediatric perioperative studies have increasingly evaluated the rate of change in pain intensity over time by conducting trajectory analyses. This type of analysis allows for an evaluation of the dynamic nature of pain intensity (or other relevant variables) across the postoperative period. For example, Sieberg et al.^7^ found five different pain trajectories after adolescent scoliosis surgery as measured by the Scoliosis Research Scale–30 (SRS-30), which included a no pain group, a pain improvement group, a short-term pain group, a delayed pain group, and a high pain group. Similarly, Ocay et al.^15^ found four trajectories with the SRS-30. Using a different pain measure (i.e., NRS), with a larger sample size, Rosenbloom et al.^13^ identified two main trajectories consisting of a high pain group and a low pain group that follow a quadratic function. Other studies using the NRS or VAS have commonly found two pain trajectories.^12,16^ Though there is heterogeneity in the pain measures used, number of groups identified, and type of trajectory analysis, most children fall into a low or mild pain group. Each of the studies also identified a smaller subset of patients classified within groups with high or severe pain or increasing pain over time, indicating the need to follow these children over time and identify predictors of this pain trajectory.

Rosenbloom et al.^13^ also evaluated pain unpleasantness through trajectory analysis to find two groups (high pain unpleasantness and low pain unpleasantness), indicating an area for further examination and replication.

## Understanding Chronic Postsurgical Pain through Chronic Pain Models

Much of the literature that examines the transition to CPSP in children does so through the lens of the biopsychosocial model. This model includes child factors, such as genetics, epigenetics, sex, presurgical pain, sleep, anxiety, and pain catastrophizing, as well as parent factors, such as parents’ cognitive appraisals of their child’s pain expression^17^ and parent pain catastrophizing.^18^ One such model is the diathesis-stress model of chronic pain and disability,^19^ which posits that individuals are more likely to develop avoidance responses and subsequent pain-related disability following a trauma (stress) if they are highly anxiety sensitive (diathesis).

The evaluation of both the biopsychosocial model and diathesis-stress model of chronic pain and disability within the context of surgery has provided mixed results, particularly as they pertain to the role of parents. For example, Pagé at al.^18^ showed that parents’ own level of pain catastrophizing 48 to 72 hours after surgery, but not their child’s, predicted child pain intensity ratings 12 months later. Rabbitts et al.^12^ found that parent catastrophizing about their child’s pain predicted late pain recovery from major surgery. Birnie et al.,^20^ however, found that child, and not parent, pain catastrophizing predicted pre- and postsurgical pain following adolescent spinal fusion surgery. Further, Rosenbloom et al.^21^ found that presurgical parent anxiety sensitivity predicted child general disability and pain-related disability 1 year after surgery. This result suggests that parents with higher levels of anxiety sensitivity may shape the development of their youth’s functional limitations in the year after surgery. Though the results are equivocal in regard to the specific parent and child constructs that predict pediatric CPSP, likely due in part to methodological factors and clinical factors (e.g., sample sizes and type of surgery), these studies suggest that certain psychological risk factors (or vulnerabilities) in both the child and the parent predispose a child to develop CPSP.

The pediatric fear avoidance model^22^ is the prevailing model within the pediatric chronic pain literature. It stems from the original cognitive–behavioral fear avoidance model of chronic pain,^23–25^ which proposes that pain may be perceived as a threat and that pain-related fears, including pain catastrophizing, dominate thought processes. These pain-related fears then lead to avoidance behaviors as well as hypervigilance to somatic symptoms, like pain. The model then proposes that avoidance and hypervigilance play roles in long-term disability and depression, which feed back into the pain experience, and thus a reiterative cycle begins. Asmundson et al.^22^ proposed that this cycle involves parents, such that parents’ or caregivers’ psychological responses to their child’s psychological pain responses directly and indirectly impact their child’s escape avoidance behaviors, which then feed back into a never-ending cycle. This model has since been adapted as the interpersonal fear avoidance model, which more fully explores the connection between parents and children in chronic pain disability.^26^

The diathesis-stress model and the interpersonal fear avoidance model have been tested, in part, over the past decade within the context of surgery and the transition from acute to chronic pain. The following sections discuss these recent findings.

### Factors Associated with Chronic Postsurgical Pain

#### Surgical Factors Associated with Chronic Postsurgical Pain

With the exception of Chidambaran et al.,^6^ who found that the duration of surgery was positively associated with 1-year chronic postsurgical pain intensity, there is overwhelming evidence that intraoperative factors, such as type of surgery, instrumentation used, and duration of anesthesia, are not associated with chronic postsurgical pain outcomes, especially when psychosocial factors are included in the statistical model.^2,7–9,11–13,15,16,18^

In adults, surgery type (e.g., amputation, thoracic surgery, Coronary Artery Bypass Graft (CABG) surgery) is a major factor in predicting postoperative pain outcomes, particularly as they relate to the development of neuropathic pain.^3^ It may be that surgery type is also a predictor in children and adolescents; however, there are a few roadblocks preventing the evaluation of the rates of neuropathic pain in pediatric surgeries compared to adult surgeries. First, the pediatric research to date has focused on select surgeries (Table 1), such as spinal fusion for scoliosis, osteotomies, and thoracotomies, that are nerve sparing. Second, it is possible that there is a reporting bias, because only one of ten studies^9^ evaluated neuropathic pain using a self-report measure (i.e., DN4). Third, it is possible that there is something protective about being a child and that puberty removes the protection from the development of neuropathic pain. Future research should examine features of neuropathic pain using validated measures or physical exam.

### Pain-related Factors Associated with Chronic Postsurgical Pain

There is substantial evidence in the adult literature that pain predicts pain.^3,27,28^ This relationship has also been shown in the pediatric literature. For example, the presence of pain before surgery,^6,8^ high doses of opioids (a proxy for more intense pain) administered in-hospital,^6^ moderate-to-severe pain intensity trajectory group membership immediately after surgery,^15^ and high levels of pain unpleasantness immediately after surgery^11^ have all been found to independently predict pain 6 to 12 months after pediatric surgery.

Given that 35% to 62% of children and adolescents report moderate-to-severe pain intensity prior to major surgery,^7,8,13^ it is important to consider prior surgical pain experiences as a potential predictor of long-term postsurgical pain outcome. It is also important to evaluate early acute postoperative pain experiences. Pagé et al.^11^ found that pain 2 weeks after surgery predicted the development of moderate-to-severe pain 1 year later. Specifically, children who had a pain score of 3 or more (on a 0–10 NRS pain intensity and NRS pain unpleasantness) 2 weeks after surgery were ~2.5 times more likely to go to develop and report moderate-to-severe CPSP 1 year later than children whose 2-week pain scores were less than 3/10. Additionally, Julien-Marsollier et al.^9^ reported that preoperative pain and acute postoperative opioid consumption predicted 12-month neuropathic pain. Though this study used a small sample size (*n* = 36), it highlights that pain predicts pain, including neuropathic pain.

### Child Psychosocial Factors Associated with Chronic Postsurgical Pain

A number of child psychosocial factors have been evaluated in the prediction of the transition from acute pain to CPSP. These factors span the domains of pain-related anxiety (pain anxiety, pain catastrophizing, fear of movement), anxiety sensitivity, pain self-efficacy, chronic pain acceptance, symptoms of posttraumatic stress disorder, symptoms of depression, and general anxiety. An important distinction has been made between the predictors of the transition to chronicity (e.g., 3–6 months after surgery) versus the maintenance (e.g., 12 months after surgery) of CPSP.

Pagé et al.^11^ found that pain unpleasantness ratings 2 weeks after surgery predicted the transition from acute to chronic pain (i.e., from 2 weeks to 6 months) but not the maintenance of CPSP (i.e., at 12 months). They also found that anxiety sensitivity, measured 2 weeks after surgery, predicted the maintenance of moderate-to-severe CPSP 6 to 12 months after surgery but not before that time. Other factors emerge when predicting long-term maintenance of CPSP and include presurgical functional disability,^13^ worse self-image,^7^ overall worse presurgical mental health,^7^ greater presurgical anxiety sensitivity,^6,11^ and poorer presurgical pain coping self-efficacy.^16^

Child pain catastrophizing, or the tendency to magnify, ruminate about, or feel helpless to pain,^29^ has been evaluated as a risk factor in most child surgical studies on pain.^6,11–13,20^ However, only one study found a significant relationship between child pain catastrophizing before surgery and pain outcomes.^20^ The weak relationship between preoperative pain catastrophizing and postoperative pain outcomes^6,11–13,20^ suggests that baseline child pain catastrophizing does not play a significant role, which is unlike the adult literature^3^ (but see Katz^30^ for a discussion of this issue).

### Parent Psychosocial Factors and Youth Chronic Postoperative Pain

The pediatric fear avoidance model of chronic pain is the prevailing model used to explain the development and maintenance of chronic pain in children.^22^ Importantly, it identifies caregiver psychological responses and caregiver pain management as having an influence on their child’s development and maintenance of chronic pain. However, to our knowledge, only six perioperative pediatric prospective studies have included parent variables, such as parent pain catastrophizing, in their modeling of the transition from acute to chronic postsurgical pain.^12,13,18,20,21,31^

Birnie et al.^20^ collected data from 167 parent and child dyads over the course of the child’s surgical experience starting prior to surgery, 4 to 6 weeks after surgery, and then again 3, 6, and 12 months after surgery. The authors evaluated the dyadic cross-sectional and longitudinal relationships between child pain, child pain catastrophizing, and parent catastrophizing about their child’s pain and concluded that baseline child, but not parent, pain catastrophizing was associated with child pain pre- and postsurgery.^20^ The authors also noted that the strength of the relationship between child pain catastrophizing and postsurgical pain is strongest in the acute postoperative period and that it fades over time. They cautioned researchers and clinicians to not classify children and parents based on their preoperative pain catastrophizing status.^20^ Their caution is line with Katz,^30^ who suggested that there may be a change in baseline pain catastrophizing due to the intense acute postoperative pain experience that intervenes between preoperative and postoperative pain catastrophizing levels. It is also possible that this change in baseline may be applicable for many factors presurgically.

### Relationship between Youth and Parent Factors in the Perioperative Period

The relationship between youth and parent measures was evaluated by Pagé et al.^18^ The authors examined correlations between child and parent pain anxiety, pain catastrophizing, and anxiety sensitivity 48 to 72 hours after surgery and 6 and 12 months after surgery. Interestingly, pain catastrophizing was significantly correlated at 6 and 12 months postsurgery but not at 48 to 72 hours postsurgery. They also noted that the magnitude of correlation coefficients between child and parent pain anxiety, pain catastrophizing, and anxiety sensitivity increased from the initial evaluation time point (i.e., 48–72 hours postsurgery) to later time points (e.g., 12 months after surgery). This suggests that youth and parents learn from each other as time in pain progresses.

## A New Model for the Transition from Acute to Chronic Pain and Disability

**Figure 1.**
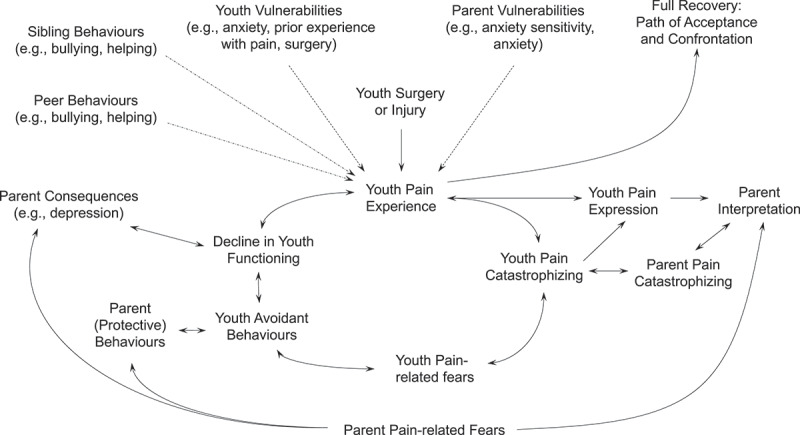
Combined pediatric diathesis-stress and interpersonal fear avoidance model of the transition from acute to chronic pain. In this model, the youth (and parents), who have certain diatheses, are exposed to a painful event (i.e., surgery) and take either a path of confrontation and acceptance of their pain (top right) or a path in which the pain is threatening and ultimately leading to disability (cycle below). In the path to pain disability, youth experience internal (i.e., pain catastrophizing, pain-related fears) and external influences (i.e., parents’ interpretations of their child’s pain expression, parent pain catastrophizing, parent protective behaviors, parent consequences) that contribute to avoidant behaviors and disability. The role of peer and sibling relationships and behaviors has not been empirically assessed as they relate to the development of CPSP in youth, but this is a fruitful avenue to explore.

As shown in Figure 1, we propose a combined diathesis-stress and interpersonal fear avoidance model to understand the transition from acute to chronic pain and disability within the context of surgery. In this new model, the diatheses include both youth (e.g., anxiety, prior poor experiences with pain/surgery, genetics) and parent (e.g., pain catastrophizing about their youth’s pain, anxiety sensitivity) vulnerabilities that can predispose youth undergoing surgery (stress) to enter a cycle of fear avoidance. These risk factors (or vulnerabilities) increase the likelihood that they will become fearful and avoidant of pain, particularly if they have multiple factors present. Once the youth undergoes surgery, they either (1) confront their pain and fear of pain and accept a certain level of pain while continuing to do what is important to them or (2) perceive the pain as threatening and enter a cycle of fear, avoidance, and pain that is influenced by, and interacts with, their parents’ interpretations and behavioral reactions to the youth’s pain expression and behavioral avoidance.

## Future Research

A logical next step in the field of pediatric CPSP is to test the proposed diathesis-stress and fear avoidance model describing the transition from acute to chronic pain. Testing this model would require a large enough sample size to evaluate each of the variables and the interactions between them. Further, using statistical analyses, such as structural equation modeling, would allow for an in-depth assessment of the interactions between variables, including their direct and indirect relationships.

As outlined in the proposed model, peer relationships are likely to play an important role in the development of CPSP. A recent systematic review and meta-analysis of cohort studies that examined the relationship between childhood bullying victimization and pain measured at least 3 months later found some evidence that bullying victimization incurs some future risk for pain, but the overall quality of evidence was very low.^32^ The research also implicates peer victimization in the perception of pain among youth.^33^ Together, the available evidence suggests a link between peer bullying victimization and later pain. This has yet to be studied in the context of CPSP.

The evidence for a relationship between peer bullying victimization in youth and subsequent pain later in life raises the question of whether sibling bullying also plays a role in the onset of pain in an affected sibling. To our knowledge, there is no published literature on this topic.^34^ However, research in other areas of mental health has shown that sibling bullying victims initially assessed at 12 years of age have increased odds of depression, suicidal ideation, and self-harm behaviors in early adulthood at the age of 24 years.^35^ Moreover, youth bullied by both siblings and peers showed even higher odds of negative mental health outcomes. These findings indicate that as pain clinicians and researchers, we must be assess the possible relationship between sibling bullying in youth and the subsequent development of chronic pain in late life. But, more generally, we should be cognizant of the potential effects, both positive and negative, that siblings have on each other’s development and, in particular, the role that brothers and sisters play in facilitating and hindering the development of chronic pain in their siblings.

Related, gender is an important social construct that deserves attention in future CPSP research. Gender can be conceptualized at the interactive, socially constructed, and regulated ways in which males and females present themselves.^36^ There is a bidirectional interaction and influence between a child’s gender and the gender of important people in the child’s life. Though measuring biological sex is important, it is also critical to evaluate the relational influence of gender on the development of pediatric CPSP (e.g., parent–child, sibling–sibling, and peer–peer).

*Resilience*, or the ability to respond effectively to risk or adversity,^37^ is a construct that has yet to be investigated in the transition from acute to chronic pain, in part because only recently has there been an available pain-specific validated measure.^38^ Acceptance of pain, psychological flexibility, and social support are all important factors in predicting adjustment to chronic illness^39,40^ and are likely important in preventing the development of CPSP. Future studies should consider including both risk and protective factors in their design.

## Conclusions

It is well known that CPSP and disability have deleterious effects. Youth pain catastrophizing, anxiety about the pain, and avoidance of activities can all lead to pain disability. However, pain and disability are not experienced in isolation. Parents’ vulnerabilities can influence their child’s pain experience, and parents’ interpretation of their child’s pain experience can have an impact on children’s pain outcomes. Future research should test the model we proposed as well as investigate the role that siblings and peers play in the development of chronic postsurgical pain. Using the proposed diathesis-stress and fear avoidance model, we can understand the hypothesized relationships between parent and youth factors and start to identify areas for intervention. Interventions may include more family-based cognitive–behavioral therapy, as starting to appear in the cancer pain literature,^41,42^ or acceptance and commitment therapy, as has been investigated in the adult transitional pain literature.^43^
